# Antimicrobial Resistance and Antimicrobial Therapy of Clinically Relevant Bacteria

**DOI:** 10.3390/antibiotics13080691

**Published:** 2024-07-25

**Authors:** Georgios Meletis, Lemonia Skoura, Efthymia Protonotariou

**Affiliations:** Department of Microbiology, AHEPA University Hospital, School of Medicine, Aristotle University of Thessaloniki, S. Kiriakidi Str. 1, 54636 Thessaloniki, Greece; mollyskoura@gmail.com (L.S.); protonotariou@auth.gr (E.P.)

Antimicrobial resistance is a major public health problem, and the World Health Organization (WHO) has warned that the current antibiotic armamentarium is not sufficient to face future challenges. There are several obstacles to the antibiotic pipeline providing new compounds at a sufficient speed [1]. As a result, at present, few novel drugs reach clinical practice, with old, formerly abandoned antimicrobials increasingly used as last-resort treatment options instead [2]. At this pace, untreatable infections could emerge at a large scale, and the world may experience dramatic situations reminiscent of the pre-antibiotic era in some cases [3]. Already, clinicians in endemic areas routinely encounter patients with infections unresponsive to the available treatments, and laboratories often report multidrug-resistant (MDR) or even pan-drug-resistant (PDR) bacteria [4]. In this context, continuous monitoring of the epidemiology of the resistance mechanisms of clinically relevant bacteria, as well as knowledge regarding the treatment options, are of great interest to healthcare professionals. This Special Issue covers manuscript submissions that further our understanding of antimicrobial resistance in clinically relevant bacteria, suggest improved methods for detecting their underlying mechanisms and provide new insights into the treatment options. Submissions on alternative or new antimicrobial compounds and the in vitro susceptibility of relevant bacteria to such compounds were especially encouraged. Based on the comments and evaluations of the reviewers and editors, eight manuscripts were selected for publication, with each one providing a unique and valuable perspective pertaining to the topics of this Special Issue.

Para-Aminosalicylic acid (PAS) is an integral anti-tuberculosis drug which requires sequential activation by two Mycobacterial compounds. Previous studies have shown that specific mutations of the *thyA* gene cause PAS resistance in *Mycobacterium tuberculosis*, but the underlying mechanisms remained unclear. In the first article in this Special Issue, Yu et al. reveal how *thyA* mutations confer PAS resistance, outlining new findings on the folate metabolism of *M. tuberculosis*.

Ceftazidime–avibactam (CAZ/AVI) is an indispensable, potentially life-saving recent addition to the treatment options for non metallo-β-lactamase-producing Gram-negative pathogens, especially KPC-producing *Klebsiella pneumoniae*. Despite its relevance, however, there are no recommendations available for its use in neonates. In the second article in this issue, Zarras et al. report a Greek case of neonatal sepsis caused by CAZ/AVI-resistant KPC-2-encoding *K. pneumoniae*, which co-harbored the *bla*_VEB-25_ gene.

Carbapenem-resistant *Acinetobacter baumannii* (CRAB) ranks high on the WHO global pathogen list and is widespread in many parts of the globe. Unfortunately, the epidemiologic situation of CRAB is poorly understood for certain countries, including Bahrain. Al-Rashed et al. shed light on this topic, demonstrating the prevalence of carbapenemases in CRAB isolated from four major hospitals within the Kingdom of Bahrain.

Due to the subjectivity of the clinical criteria and the low discriminative power of the diagnostic tests used, diagnosing ventilator-associated pneumonia (VAP) early remains a challenge. Therefore, novel biomarkers are urgently needed. In the fourth article in this issue, Sdougka et al. evaluated five host inflammatory biomarkers for the early diagnosis of VAP in critically ill children.

Promptly detecting carbapenemases in clinical and/or surveillance isolates recovered from healthcare settings such as hospital wards or ICUs is crucial for the timely implementation of infection control measures. In the fifth article in this issue, Tychala et al. evaluated the effectiveness and benefits of replacing the traditional phenotypic methods with a rapid immunochromatographic assay for Enterobacterales and *Pseudomonas aeruginosa*.

Efflux pumps represent an important bacterial mechanism conferring multi-drug resistance in Gram-negative bacteria; however, they have been less frequently investigated than enzymatic resistance determinants. In their study, Quddus et al. report intriguing efflux pump mutations in *P. aeruginosa* isolates from Pakistan.

Despite polymyxins commonly being used as a last-resort treatment for *A. baumannii* and *K. pneumoniae*, polymyxin resistance is on the rise worldwide. In an effort to diversify the treatment options for these stubborn sources of infection, Mantzana et al. assessed the in vitro synergistic activity of using specific antimicrobial combinations against carbapenem-resistant and colistin-resistant *A. baumannii* and *K. pneumoniae*.

The increasing resistance of *Salmonella* spp. to antimicrobials has galvanized the search for new alternatives, including natural compounds such as curcumin. In the final article in this Special Issue, Zermeño-Ruiz et al. aimed to verify the antibacterial activity of curcumin in relation to the growth rate, virulence and pathogenicity of *Salmonella enterica* serovar Typhimurium. Based on their results, the authors suggest reconsidering the indiscriminate use of curcumin in response to outbreaks of pathogenic Gram-negative bacteria.

Overall, the articles included in this Special Issue of Antibiotics offer new data on the antimicrobial resistance and epidemiology of MDR bacterial infections of key clinical importance, as well as suitable therapeutic options for their treatment. Hopefully, these contributions will both practically benefit the readership and stimulate further research in the field of antimicrobial resistance.
